# Neuromuscular dysfunction in patient-derived FUS^R244RR^–ALS iPSC model via axonal downregulation of neuromuscular junction proteins

**DOI:** 10.1093/narmme/ugaf005

**Published:** 2025-03-12

**Authors:** Nicolai von Kügelgen, Katarzyna Ludwik, Samantha Mendonsa, Christine Römer, Erik Becher, Laura Breimann, Mara Strauch, Tommaso Mari, Sandrine Mongellaz, Binyamin Zuckerman, Fatima Efendic, Nina Grexa, Anna Oliveras-Martinez, Andrew Woehler, Matthias Selbach, Vincenzo La Bella, Igor Ulitsky, Marina Chekulaeva

**Affiliations:** Max-Delbrück-Center for Molecular Medicine in the Helmholtz Association (MDC), Berlin Institute for Medical Systems Biology (BIMSB), Non-coding RNAs and mechanisms of cytoplasmic gene regulation, Berlin 10115, Germany; Max-Delbrück-Center for Molecular Medicine in the Helmholtz Association (MDC), Berlin Institute for Medical Systems Biology (BIMSB), Non-coding RNAs and mechanisms of cytoplasmic gene regulation, Berlin 10115, Germany; Max-Delbrück-Center for Molecular Medicine in the Helmholtz Association (MDC), Berlin Institute for Medical Systems Biology (BIMSB), Non-coding RNAs and mechanisms of cytoplasmic gene regulation, Berlin 10115, Germany; Max-Delbrück-Center for Molecular Medicine in the Helmholtz Association (MDC), Berlin Institute for Medical Systems Biology (BIMSB), Non-coding RNAs and mechanisms of cytoplasmic gene regulation, Berlin 10115, Germany; Max-Delbrück-Center for Molecular Medicine in the Helmholtz Association (MDC), Berlin Institute for Medical Systems Biology (BIMSB), Non-coding RNAs and mechanisms of cytoplasmic gene regulation, Berlin 10115, Germany; Harvard Medical School, Department of Genetics, Boston02115, MA, United States; Max-Delbrück-Center for Molecular Medicine in the Helmholtz Association (MDC), Berlin Institute for Medical Systems Biology (BIMSB), Non-coding RNAs and mechanisms of cytoplasmic gene regulation, Berlin 10115, Germany; Max Delbrück Center for Molecular Medicine in the Helmholtz Association (MDC), Proteome Dynamics, Berlin 13125, Germany; Max-Delbrück-Center for Molecular Medicine in the Helmholtz Association (MDC), Berlin Institute for Medical Systems Biology (BIMSB), Non-coding RNAs and mechanisms of cytoplasmic gene regulation, Berlin 10115, Germany; Weizmann Institute of Science, Department of Biological Regulation and Molecular Neuroscience, Rehovot7610001, Israel; Max-Delbrück-Center for Molecular Medicine in the Helmholtz Association (MDC), Berlin Institute for Medical Systems Biology (BIMSB), Non-coding RNAs and mechanisms of cytoplasmic gene regulation, Berlin 10115, Germany; Max-Delbrück-Center for Molecular Medicine in the Helmholtz Association (MDC), Berlin Institute for Medical Systems Biology (BIMSB), Non-coding RNAs and mechanisms of cytoplasmic gene regulation, Berlin 10115, Germany; Humboldt University of Berlin,10115, Germany; Max Delbrück Center for Molecular Medicine in the Helmholtz Association (MDC), Berlin Institute for Medical Systems Biology (BIMSB), BIMSB Light Microscopy platform, Berlin 10115, Germany; Max Delbrück Center for Molecular Medicine in the Helmholtz Association (MDC), Berlin Institute for Medical Systems Biology (BIMSB), BIMSB Light Microscopy platform, Berlin 10115, Germany; Max Delbrück Center for Molecular Medicine in the Helmholtz Association (MDC), Proteome Dynamics, Berlin 13125, Germany; University of Palermo, Department of Experimental Biomedicine and Advanced Diagnostics, ALS Clinical Research Center, Laboratory of Neurochemistry, Palermo 90133, Italy; Weizmann Institute of Science, Department of Biological Regulation and Molecular Neuroscience, Rehovot7610001, Israel; Max-Delbrück-Center for Molecular Medicine in the Helmholtz Association (MDC), Berlin Institute for Medical Systems Biology (BIMSB), Non-coding RNAs and mechanisms of cytoplasmic gene regulation, Berlin 10115, Germany

## Abstract

Amyotrophic lateral sclerosis (ALS) is a neurodegenerative condition characterized by the progressive degeneration of motor neurons (MNs), ultimately resulting in death due to respiratory failure. A common feature among ALS cases is the early loss of axons, pointing to defects in axonal transport and translation as initial disease indicators. ALS is associated with mutations in RNA-binding proteins, such as FUS (Fused in Sarcoma). Here, we established a FUS^R244RR^–ALS hiPSC-derived model that recapitulates the MN survival and muscle contractility defects characteristic of ALS patients. Analysis of the protein and mRNA expression profiles in axonal and somatodendritic compartments of ALS-afflicted and isogenic control MNs revealed a selective downregulation of proteins essential for the neuromuscular junction function in FUS–ALS axons. Furthermore, analysis of FUS CLIP and RIP data showed that FUS binds mRNAs encoding these proteins. This work shed light on the pathogenic mechanisms of ALS and emphasized the importance of axonal gene expression analysis in elucidating the mechanisms of neurodegenerative disorders.

## Graphical Abstract

**Graphical Abstract:**
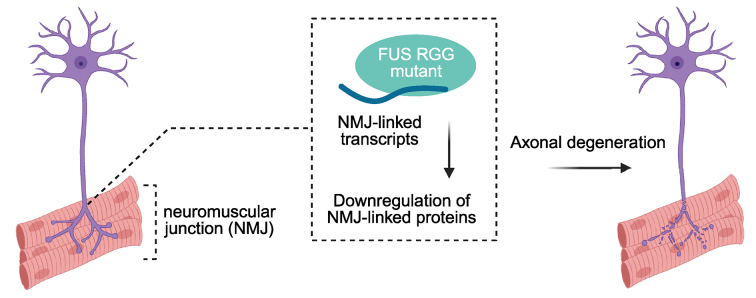

## Introduction

Neurodegeneration disorders are devastating diseases, predicted to become the second leading cause of death by 2040 due to population aging [1]. Amyotrophic lateral sclerosis (ALS), also known as Lou Gehrig’s disease, arises from the progressive loss of motor neurons, leading to paralysis and respiratory failure, with no cure currently available. ALS cases of different etiology share defects in RNA metabolism, particularly in splicing and axonal mRNA transport (reviewed in [2]). Notably, mutations in the RNA-binding proteins (RBPs) TAR DNA-binding protein 43 (TDP43) and Fused in Sarcoma (FUS) are known causes of ALS, and pathologic TDP43 inclusions are found in the brain and spinal cord of 97% of ALS patients, suggesting that defects in RNA metabolism are a common feature in ALS. To date, most therapeutic approaches are aimed at protecting neuronal cell bodies from degeneration. However, in ALS axonal degeneration occurs prior to the death of neuronal cell bodies (soma) and correlates with the onset of functional decline (reviewed in [3]). The primary role of axonal degeneration in disease progression is supported by postmortem studies [4], mouse models [5], and electrophysiological observations [6]. Therefore, investigating local axonal gene expression in ALS is critical for future research.

Additionally, it is crucial to acknowledge the limitations of mouse models in neurodegenerative research. Although treatments in mouse ALS models have spurred over 50 clinical trials, none have translated into successful human therapies, pointing to fundamental differences in disease biology between humans and mice [7]. Furthermore, mouse models fall short of representing sporadic ALS, which accounts for over 90% of cases [8]. Hence, there is an imperative need to develop ALS models that reflect the genetic background of patients. Since the disease targets motor neurons (MNs), generating human neurons with the patient’s genetic background is feasible only through differentiation of patient-derived induced pluripotent stem cells (iPSCs) into MNs.

In this context, we tackled both aforementioned challenges. Using FUS^R244RR^–ALS patient fibroblasts, we established an iPSC line alongside an isogenic control where the FUS^R244RR^ mutation was corrected. These iPSC lines were then differentiated into spinal MNs through direct programming [9–14]. Our FUS^R244RR^–ALS model successfully replicated the MN survival and muscle contractility impairments seen in ALS patients. Previously, we developed a technique for isolating neuronal subcellular compartments—specifically, cell bodies and neurites—for omics analyses (referred to as spatial omics, [15–19]). We adapted this approach to iPSC-derived MNs, separating their axonal and somatodendritic fractions for comprehensive omics analyses. Analysis of the protein and mRNA expression levels in axonal and somatodendritic compartments of ALS and control MNs identified a preferential downregulation of proteins critical for neuromuscular junction (NMJ) functionality in FUS–ALS axons. Additionally, our examination of FUS CLIP and RIP datasets showed a marked enrichment of direct FUS targets among the downregulated proteins. This work illuminates the pathogenic mechanisms of ALS and emphasizes the crucial role of axonal gene expression analysis in understanding neurodegenerative disorders.

## Materials and methods

### Cell line generation

The stem cell line (Hues-3 HB9-GFP) was obtained from Harvard cell line collection [20]. WT hiPSC line (BIH005-A) was obtained from the BIH-MDC stem core facility (https://hpscreg.eu/cell-line/BIHi005-A). ALS patient fibroblasts were collected at the ALS Clinic (Palermo) and reprogrammed into human induced pluripotent stem cells (hiPSC) by Applied Stem Cell and edited into wild-type by Axol. To generate cell lines for differentiation into induced MNs (iMNs), the construct with the inducible NIL cassette [14] (Addgene #105841) was transfected into these lines using Lipofectamine 3000 and stably inserted into using TALENs (Addgene #62197 and #62196). To generate the cell line for differentiation into muscle cells (myoD-hiPSC), the construct (Addgene #105841) was modified to replace the NIL cassette with myoD coding sequence and stably integrated into BIH005-A using the same procedure.

### Cell culture and iMN differentiation protocol

hiPSC lines and Hues-3 line were grown in E8 medium, which was exchanged daily, and passaged before they reached full confluency. Differentiation was performed largely as described previously [14]. For differentiation, cells were split with accutase from ∼80% confluent culture into Geltrex (Thermo A1413302) coated six-well plates with 1–1.5 million cells per well in E8 with 10 μM ROCK inhibitor Y-27632 (Tocris 1254). On day 1, the differentiation medium was replaced with induction medium (IM), which consisted of DMEM/F12 w/HEPES (Thermo 11330-032), N2 supplement (100×, Thermo 17502-048), nonessential amino acid (100×, Thermo 1140050), Glutamax (100×, Thermo 35050-061), ROCK inhibitor (10 μM), doxycycline (2 μg/ml), and Compound E (0.1 μM, Enzo Life ALX-270-415-C250). On day 3, MN progenitors were replated using accutase, and the medium was exchanged to IM supplemented with 5-fluoro-2'-deoxyuridine (FUDR, 8 μM, Sigma 50-91-9). Replating was done in accordance with the planned experiments: to Geltex-coated six-well plates for isolation of RNA or protein from total cells, and to Geltrex-coated filter membrane inserts (pore size: 1.0 μm PET membrane for six-well plate Millicell PLRP06H48, Millipore) for isolation of RNA or protein from subcellular compartments or imaging on filters, to acid-treated and poly-D-lysine (100 μg/ml) and laminin (5 μg/ml) coated glass slides for immunofluorescence (IF) and imaging, or to poly-D-lysine (100 μg/ml) and laminin (5 μg/ml) coated CytoView MEA 48-plates (AXION Biosystems, M768-tMEA-48W) for direct electrophysiology measurements. On day 4, medium was exchanged to MN media (MM), which consisted of Neurobasal medium (Thermo 21103-049), B27 (50×, Thermo 17504-044), N2 (100×, Thermo 17502-048), nonessential amino acid (100×, Thermo 1140050), Glutamax (100×, Thermo 35050-061), Laminin (1 μg/ml), 10 ng/ml of BDNF (Peprotech 450-02), CNTF (Peprotech 450-13), and GDNF (Peprotech 450-10), supplemented with doxycycline (1 μg/ml), Compound E (0.1 μM, Enzo Life ALX-270-415-C250), and FuDR (8 μM, Sigma 50-91-9). From then on, MM was exchanged every 2–3 days, and FUDR, Compound E, and doxycycline were added to MM only until day 9.

### Harvesting of RNA and protein material

Protein material was harvested from filter membranes using 8 M urea, 0.1 M Tris–HCl, pH 7.5, and RNA material, using TRIfast (PeqLab 30-2010). For a detailed description of harvesting material from filter membranes, see our prior work [17].

### hiPSC-derived neuromuscular culture

Myogenic differentiation was performed using published protocols [21–24] with some modifications. Specifically, the myoD-hiPSC line, which contains myoD under a doxycycline-inducible promoter, was split using accutase. One million cells were plated per well of a Geltrex-coated six-well plate in E6 medium (Thermo A1516401) supplemented with 10 μM ROCK inhibitor Y-27632 (Tocris 1254).

The next day (day 2), the medium was changed to E6 with 1 μg/ml doxycycline. On day 3, the cells were split using accutase, and 40 000 cells were plated per well of a Geltrex-coated 96-well square glass bottom plate (ibidi 89627) in KSR/αMEM medium. This medium consisted of 5% KnockOut Serum Replacement (KSR, Thermo 10828028), 55 mM 2-mercaptoethanol, 1× penicillin/streptomycin (Nacalai Tesque 2625384), and minimum essential medium α (MEMα; Thermo 12561056), and was additionally supplemented with 10 μM ROCK inhibitor and 1 μg/ml doxycycline.

The next day, the medium was changed to KSR/αMEM with 1 μg/ml doxycycline. Medium changes continued every second day until day 7. On day 8, the medium was switched to HS/DMEM, which included horse serum (Sigma H1138), DMEM high glucose (Thermo 11960085), 1× GlutaMAX (Thermo 35050-061), 55 mM 2-mercaptoethanol, and 1× penicillin/streptomycin, 10 ng/ml IGF-1 (PeproTech 100-11), and was additionally supplemented with 1 μg/ml doxycycline. Medium changes were performed every second day.

On day 14, 25 000 MN progenitors, generated via direct programming as described above, were co-plated per well in MM, supplemented with 1 μg/ml doxycycline and 10 ng/ml Insulin-like Growth Factor 1 (IGF-1). The medium was exchanged twice a week, with doxycycline included until day 20. Contractions were recorded at 6 weeks of myoD culture.

### Electrophysiology measurements using axion MEA plate

For electrophysiology measurements of iMN at day 21, the Maestro Edge multi-electrode array (MEA) system (AXION Biosystems) with CytoView MEA 48-plates (AXION Biosystems, M768-tMEA-48W) was used, and raw data were processed using AxIS Navigator software (version 2.0.4). Individual voltage spike events were extracted from the raw waveform of an exemplary electrode were analysed using the Spike Detector processing tool with a detection threshold of 5.5xSTD and then averaged for plotting.

### Lactate dehydorgenase assay

The lactate dehydorgenase (LDH) assay was conducted using the CyQuant LDH Cytotoxicity Assay Kit (Thermo Fisher, C20300), according to the manufacturer’s instructions, with some modifications. In brief, iMNs were seeded onto a Geltrex-coated 96-well plate at a density of 187 500 cells/cm² and cultured following the iMN differentiation protocol. From day 21 onwards, media was changed weekly following the LDH measurement. LDH released by plasma-membrane-damaged iMNs into the media was quantified spectrophotometrically by measuring absorbance at 490 nm, with 680 nm used as the reference wavelength. Measurements from wells containing only media on the same plate served as a reference value.

### Immunofluorescence

For immunostaining, cells were grown on coverslips or ibidi plates as described above. Before staining, cells were fixed with 4% paraformaldehyde for 20 min and permeabilized with 75% EtOH or 0.2% Triton X-100 in phosphate-buffered saline (PBS) for 10 min. For NMJ staining, 0.3% Triton X-100 in PBS was applied for 20 minutes. After blocking with 10% bovine serum albumin (BSA) in PBS for 60 min, cells were probed with the respective primary antibodies in 3% BSA in PBS overnight at 4°C, washed with 3% BSA in PBS, and incubated with secondary antibodies for 1 h before mounting them with ProLong Gold with DAPI (Cell Signaling). The following primary antibodies were used: α-β-III-tubulin (1:200, Sigma–Aldrich T2200), α-MAP2 (1:1.000, SYSY 188004), α-HB9 (1:25, DSHB 81.5C10), α-GFP (1:500, Abcam ab183734), α-neurofilament (1:5000, Biolegend BLD-822601; 1:1000, Biolegend SMI312), α-synapsin (1:500, Merck AB1543), and α-myosin heavy chain (MyHC, 1:20, DSHB A4.1025). To visualize NMJs, BTX-488 (1:100, Invitrogen B13422) was used. Images were taken using a Leica TCS SP8 confocal microscope with a ×40 or ×63 objective.

### Puro-PLA and image analysis

Detection of newly synthesized proteins by puro-PLA was performed as described previously [25]. Briefly, iMNs were incubated with 1 mg/ml puromycin for 15 min, washed quickly in PBS, and fixed with 4% PFA in PBS for 10 min at RT. Cells were washed twice in PBS and permeabilized with 0.2% Triton X-100 in PBS for 10 min at RT. Puro-PLA was performed using Duolink reagent, antibodies α-EPHA4 (1:100, Invitrogen PA514578), α-puromycin (1:200, Kerafast 3RH11), rabbit PLA^plus^, and mouse PLA^minus^ probes, according to manufacturer’s recommendations except that antibody dilution solution was replaced by 5% BSA in PBS. iMNs were immunostained with α-NF (1:1000) and mounted in Duolink *in situ* mounting medium. Images were acquired using a 40× oil objective on a Leica SP8 FALCON confocal microscope.

The image analysis pipeline is adapted from a previously published workflow [19] with the following adaptations. Max projections of the anti-Neurophillament and Puro-PLA images were generated using a Fiji (ImageJ) macro [26]. Masks for the neurites were created using LABKIT [27] and were manually corrected to represent either 30 or 70 μm of the main neurites or the soma. The puro-PLA spots were detected and quantified in 2D max projections of the images using the RS-FISH Fiji plugin [28]. The detections were subsequently filtered using the binary neurite and soma masks in Python. The puro-PLA detections were normalized by the area of the neurite masks or soma masks. The pipeline can be found at: https://github.com/LauraBreimann/ALS_puro-PLA

### Electrophysiology calcium imaging

For calcium imaging experiments day 20 iMNs plated on 35-mm glass bottom imaging dish were loaded with 5 μM CalBryte520 AM (AAT Bioquest) and 0.02% pluronic acid in 50% Tyrodes buffer (pH 7.4, 120 mM NaCl, 2.5 mM KCl, 10 mM HEPES, 10 mM glucose, 2 mM CaCl_2_, 1 mM MgCl_2_, and osmolarity adjusted with sucrose to MM) and 50% MM for 30 min at 37°C. All media was exchanged to Tyrodes buffer immediately before imaging. To induce action potentials (APs), a custom-made electrical stimulation chamber containing two electrodes was placed inside the iMN culture dish. The dish was mounted on a Dragonfly spinning disk microscope (Andor, Oxford Instruments) and connected to a pulse generator (A-M Systems Model 2100). After 5 s of baseline recording, iMN were stimulated by applying five field pulses at 9 s intervals, 1 ms width, and 20 V amplitude, followed by imaging up to 2.5 min to detect spontaneous firing. Imaging was performed using (488 nm with an exposure time of 100 ms) illumination in a 10× objective, and intracellular calcium dynamics were recorded at a frequency of 10 Hz. The post-acquisition analysis was performed using custom Matlab scripts, which normalized changes in fluorescence to the pre-stimulus baseline fluorescence. The last AP was plotted for each region of interest (ROI), and the average of individual ROIs was calculated.

### Western blot

For the western blot, 2.5 μg protein were separated on a 10% SDS–PAGE and then transferred to a polyvinylidene fluoride (PVDF) membrane. The following primary antibodies were used to probe the membrane: α-neurofilament (1:10 000, Biolegend BLD-822601), α-synapsin (1:500, Merck AB1543), α-synaptophysin (1:250, Life Technologies PA11043), α-chat (1:100, Merck AB144P), α-β-actin (1:4.000, Sigma A2228), α-GAP43 (1:1000, Santa Cruz sc-10786), α-RNA-PolII (1:200 000, Biolegend MMS-128P), and α-H3 (1:3000, Thermo PA5-31954).

### qPCR

RNA from total cells or separated compartments was treated with RQ1 Dnase and reverse-transcribed using the Maxima first strand cDNA synthesis kit (Thermo Fisher). Quantitative polymerase chain reaction (qPCR) was performed using sensiFAST SYBR No ROX qPCR kit (Bioline) and a CFX96 Real-Time PCR system (Bio-Rad). Expression level changes were calculated using ΔΔCt method with β-actin as a reference RNA for total cells and expression normalised to day 1 timepoint or with rRNA as a reference RNA for separated compartments. The following primers were used: Actb-fw (GAGCACAGAGCCTCGCCTTT), Actb-rev (ACATGCCGGAGCCGTTGTC), rRNA-fw (AAACGGCTACCACATCCAAG), rRNA-rev (CCTCCAATGGATCCTCGTTA), NANOG-fw (ATGCCTCACACGGAGACTGT), Snord15b-fw (CAGTGATGACACGATGACGA), Snord15b-rev (AGGACACTTCTGCCAAAGGA), Rab13 (Primerbank ID 34850075c1), Kif1c (Primerbank ID 291327508c2), Kif5a (Primerbank ID 45446748c1), and Gap43 (Primerbank ID 194248055c1).

### Mass spectrometry sample preparation

Samples for proteomics were harvested from total cells (one six-well per replicate for time points day 1, 2, 3, and 4 and two wells per replicate for time points day 7, 14, and 21) and separated compartments (two six-well membrane filters per sample) as described above and lysed in 8 M urea lysis buffer and processed for in-solution protein digestion [29]. All samples were prepared in triplicates. Briefly, disulfite bridges were reduced by treatment with 5 mM dithiothreitol (DTT) for 1 h at room temperature and subsequently alkylated with 5 mM iodoacetamide (1 h, room temperature in the dark). Then samples were diluted with 50 mM Tris to reach a final urea concentration of 2 M, pre-digested with LysC (Wako Chemicals) at a 1:50 (w:w) ratio for 2 h, then trypsin (Promega) was added at 1:50 (w:w) ratio, and samples were digested overnight at room temperature. Proteolytic digestions were stopped by acidification with the addition of formic acid (FA) to a final concentration of 1%. Digests were acidified with FA and centrifuged (20 000 *× g*, 15 min) to remove the precipitated urea. The resulting peptides were de-salted via stop-and-go extraction [30]. Two disks of C18 (3M Empore) material were inserted in a 200 μl pipette tip, activated via washes with methanol followed by washes with 50% acetonitrile (ACN) and 0.1% FA and only 1% FA. Samples were loaded onto the Stage-tips, and the retained peptides were washed twice with 0.1% trifluoroacetic acid (TFA), followed by a wash with 1% FA. Finally, peptides were eluted from the C18 material with a solution of 50% ACN/0.1% FA and resuspended in mobile phase A (0.1% FA and 3% ACN in water) prior to mass spectrometric analysis.

### Liquid chromatography coupled with tandem mass spectrometry

Approximately 1 μg of peptides for each sample was online-separated on an EASY-nLC 1200 (Thermo Fisher Scientific) and acquired on a Q-Exactive HFx (Thermo Fisher Scientific), and samples were run with a randomized order. Peptides were separated on a fused silica, 25 cm long column packed in-house with C18-AQ 1.9 μm beads (Dr. Maisch Reprosil Pur 120) kept at a temperature of 45°C. After equilibrating the column with 5 μl mobile phase A, peptides were separated with a 250 μl/min flow on a 214 min gradient: mobile phase B (0.1% FA and 90% ACN) increased from 2% to 30% in the first 192 min, followed by an increase to 60% in the following 10 min, to then reach 90% in 1 min, which was held for 5 min. The mass spectrometer was operated in data-dependent acquisition, with MS1 scans from 350 to 1500 *m*/*z* acquired at a resolution of 60 000 (measured at 200 *m*/*z*), maximum injection time (IT) of 10 ms, automatic gain control (AGC) target value of 3 × 10^6^, and recording in profile mode. The 20 most intense precursor ion peaks with charges from +2 to +6 were selected for fragmentation, unless present in the dynamic exclusion list (30 s). Precursor ions were selected with an isolation window of 1.3 *m/z*, fragmented in an HCD cell with a normalized collision energy of 26%, and analyzed in the detector with a resolution of 15 000 *m/z* (measured at 200 *m/z*), AGC target value of 10^5^, maximum IT of 22 ms, and recording in centroid mode.

### Proteomics data analysis

RAW files were analyzed using MaxQuant [31] version 1.6.3.4 using MaxLFQ as a quantification method [32]. The MS scans were searched against the human Uniprot databases (Jan 2020) using the Andromeda search engine with FDR calculated based on searches on a pseudo-reverse database and set to 0.05. The search included as fixed modifications carbamidomethylation of cysteine and as variable modifications methionine oxidation, N-terminal acetylation, and asparagine and glutamine deamidation. Trypsin/P was set as a protease for *in silico* digestion. All samples (time points and soma/neurite comparisons) were analyzed in the same MaxQuant session. The resulting protein groups were filtered for protein contaminants, hits in the reverse database, only identified by modified site and identified by less than two peptides, of which one was unique. In the analysis of the time course experiment, we also filtered out proteins that were not identified in all replicates in at least one time point. The remaining missing values were imputed by randomly selecting values from a normal distribution with a 30% the standard deviation and shifted downwards by 1.8 standard deviation units [33].

For analysis of expression levels imputed label-free quatification (LFQ) values were log-transformed, averaged between replicates and protein identifiers mapped to ensembl gene IDs. For heatmaps values were also scaled to *z*-scores (number of standard deviations from the mean) for each protein across all time points.

In the analysis of the cellular compartments, proteins were selected when present in all replicates of one cellular compartment, and the remaining missing values were imputed as described above. Proteins differentially expressed between soma and neurite were evaluated with a modified Student’s *t*-test [33] with an S0 value of 1. Statistical analysis was done with R (v3.6.3). *P*-values were set to 1 for proteins detected in less than two samples of any compartment.

### RNA-seq libraries

Hundred nanograms of total RNA obtained from either total cells or separated compartments was used for library preparation with the Truseq stranded total library prep kit (Illumina 20020596) or Truseq stranded mRNA library prep kit (Illumina 20020595), according to the manufacturer’s recommendation. Each library was prepared in triplicate and sequenced on an Illumina NextSeq 500 sequencer with single-end 150 bp reads.

### RNA-seq data analysis

All fastq files were trimmed, mapped, and counted using the PiGx RNAseq pipeline [34] version 0.0.10. For analysis of expression values raw counts generated by salmon were scaled to transcripts-per-million (TPM) and genes with average TPM values below one were removed before log-transformation of TPM values. Some of our individual samples that did not cluster with replicates in PCA were removed before any further analysis. For heatmaps, TPM values were also scaled to *z*-scores (number of standard deviations from the mean) for each gene across all timepoints. Differential expression between compartments was performed using the PiGx pipeline, but only genes with TPM > 1 in at least two replicates were retained for analysis.

For alternative splicing analysis, STAR alignment of the RNA-seq data was fed through the LeafCutter pipeline [35] with default parameters. Events with *P* < 0.05 were considered as significant.

Public available datasets were obtained from the following accessions: GSE108094 (control samples SRR6376956-59 [36]), GSE69175 (control samples SRR2038215-16 [37]), GSE121069 (human control SRR7993125-37 [38]), SRP064478 (healthy controls SRR2558717-24 [39]), GSE41795 (positive samples SRR606335-36 [40]), and GSE66230 (SRR1814073,75-76,78,79-80 [41]). Fastq files were processed using the same PiGx workflow as before. Raw count values were extracted from supplementary files of Maciel *et al.* [42] and Rotem *et al.* [43]. Variance stabilizing transformation was applied to raw counts from all datasets for those genes with per-dataset average TPM values >1 using DESeq2. PCA was then performed on median values from the total or soma compartments of each dataset.

### Gene ontology analysis

Gene Ontology (GO) analysis was performed using the gprofiler2 R package [44] call to web-interface (June 2020) on genes that had significant enrichment (p.adj < 0.05) with |log2fd| > 1 in either soma or axon compartment on both RNA and protein (MS) level. GO terms enriched in the localized gene set from both compartments were then filtered for a maximum of 1000 genes per term and at least 25 overlapping localized transcripts/proteins with each term and further based on the graph of GO term relationships: for each connected sub-tree of enriched terms (nodes), only the lowermost (no enriched direct daughter terms) and uppermost (no direct enrich parent terms) were retained (GO release 2020-06). For each such selected GO term protein localization values (log2fd) from all genes annotated as members of this term were used for the generation of figures.

### Cell type deconvolution

Raw fastq files for single cells as well as their AP type classification were provided by Bardy *et al.* [45]. Files were processed using PiGx RNAseq pipeline, and raw counts from salmon were imported using tximport. Seurat was used to process (CreateSeuratObject, parameters: min.cells = 3) and normalise counts, and only cells with from AP type 1-5 were used, and types 1-3 were grouped together. Differentially expressed marker genes between AP type groups (1-2-3, 4, and 5) were then calculated using FindAllMarkers (parameters: logfd.threshold = 0.25 and min.pct = 0.25).

All markers identified this way were used as signature markers for cell type deconvolution of total cell time points day 4 to day 21 with CIBERSORTx (non-default signature matrix settings: 25-300 barcode genes; single cell min. expression 1, replicates 0 and sampling 0; 500 permutations used for statistical analysis in cell fraction imputation [46].

## Results

### Efficient and rapid differentiation of human stem cells into motor neurons via direct reprogramming

**Figure 1.**
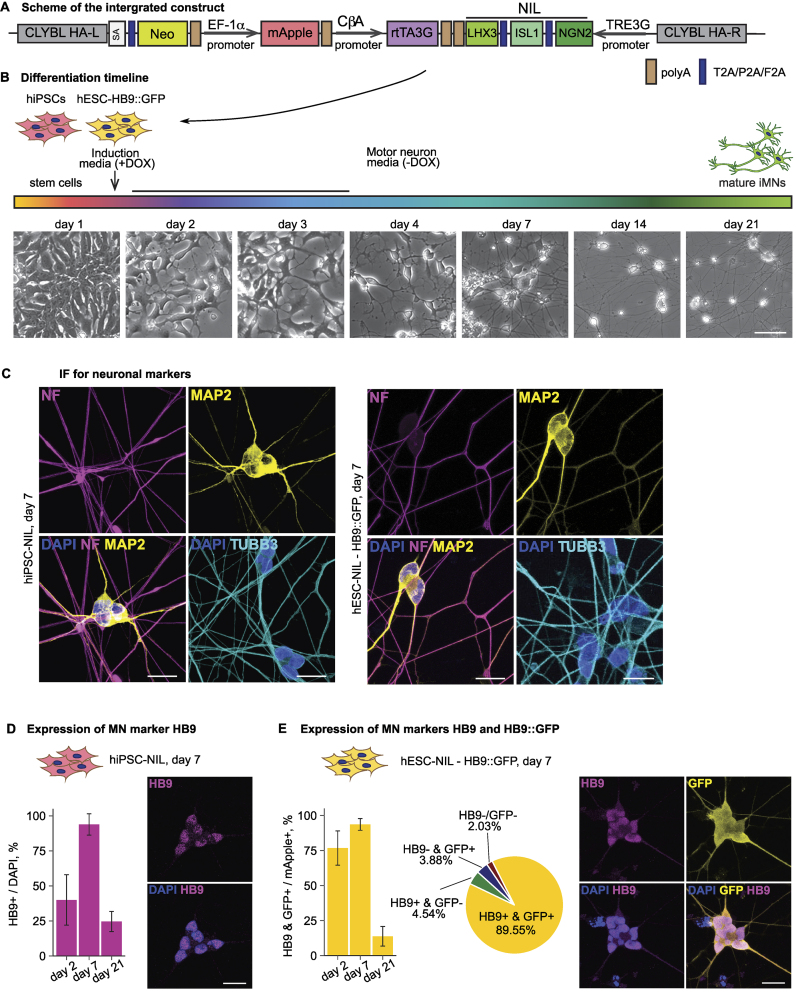
Direct programming is an efficient and robust protocol for the generation of MNs from hiPSCs and hESCs. (**A**) A scheme of the homology mediated repair template for insertion of NGN2-ISL1-LHX3 (NIL) cassette into CLYBL genetic locus. CbA: chicken β-actin; SA: splice acceptor; HA L/R: homology arm left/right; Neo: neomycin resistance gene. (**B**) Differentiation protocol time course with exemplary images (Hues-3 line) illustrating morphological changes of the cells; DOX: doxycycline; iMN: human-induced MN; scale bar: 100 μm. (**C**) iMNs express neuronal (TUBB3, cyan), dendritic (MAP2, yellow), and axonal (NF, magenta) markers. iMNs derived from hiPSC-NIL (left) and hESC-NIL-HB9::GFP (right) on day 7 are shown; scale bar: 20 μm. (**D** and **E**) Direct programming protocol produces a highly homogeneous (∼90%) MN culture. For the hiPSC-NIL differentiation, the percentage of HB9 positive cells and the exemplary IF images at day 7 are shown in panel (D). For hESC-NIL differentiation, the percentages of HB9 and GFP positive cells and the exemplary IF images at day 7 are shown in panel (E). HB9: magenta, GFP: yellow, DAPI: blue, scale bar: 20 μm.

To generate MNs from human stem cells, we relied on induced expression of transcription factors specific for MNs: NGN2, ISL1, and LHX3 [9–14] (Fig. 1A). The resulting MNs have been referred to as iMNs, iNIL (for induced NGN2, ISL1, and LHX3), or i^3^LMNs (for induced 3 factors lower MNs). In particular, we integrated a construct expressing these factors under the doxycycline-inducible promoter into the safe-harbor CLYBL locus [14] into human stem cell lines. As parental stem cell lines, we used human iPSC and embryonic stem cell lines (HuES-3 HB9::GFP, [20]) to generate NIL-hiPSC and NIL-hESC lines, correspondingly (see “Materials and methods” section for details). In addition to the transcription factors, the integrated construct constitutively expresses the reverse tetracycline transactivator (rtTA) and a fluorescent marker mApple.

Induction of resulting NIL-hiPSC and NIL-hESC lines with doxycycline resulted in neuronal differentiation (Fig. 1B). Cells started to change their morphology from day 2 (MN precursors), and long projections (neurites or neuronal processes) extended from the cell body already by day 7 (early MNs). Neurites continued to elongate up to day 21 (mature MNs).

We confirmed the neuronal identity on day 7 of differentiation by immunostaining with neuronal (Fig. 1C, TUBB3), dendritic (MAP2), and axonal [neurofilament (NF)] markers. To validate the MN identity of resulting neurons and evaluate the efficiency of differentiation, we used immunostaining and imaging to analyze the expression of HB9, a transcription factor characteristic of early MNs [47]. On day 7, >90% of cells were HB9-positive, both in the case of NIL-hiPSC- (Fig. 1D) and NIL-hESC-derived neurons (Fig. 1E). The NIL-hESC line also carries the green fluorescent protein (GFP) under the control of the *HB9* promoter: in these cells, GFP is only expressed when the *HB9* gene is active. Hence, GFP expression serves as an easily detectable visible marker for differentiation efficiency. Almost 90% of the NIL-hESC-derived neurons expressed both GFP and endogenous HB9 (Fig. 1E). These data show that the expression of MN-specific transcription factors allows much higher differentiation efficiency (∼90%) than protocols based on small molecules (<50% [48–51], reviewed in [52]).

### iMNs display expected differentiation patterns and are transcriptomically similar to primary mature motor neurons

**Figure 2.**
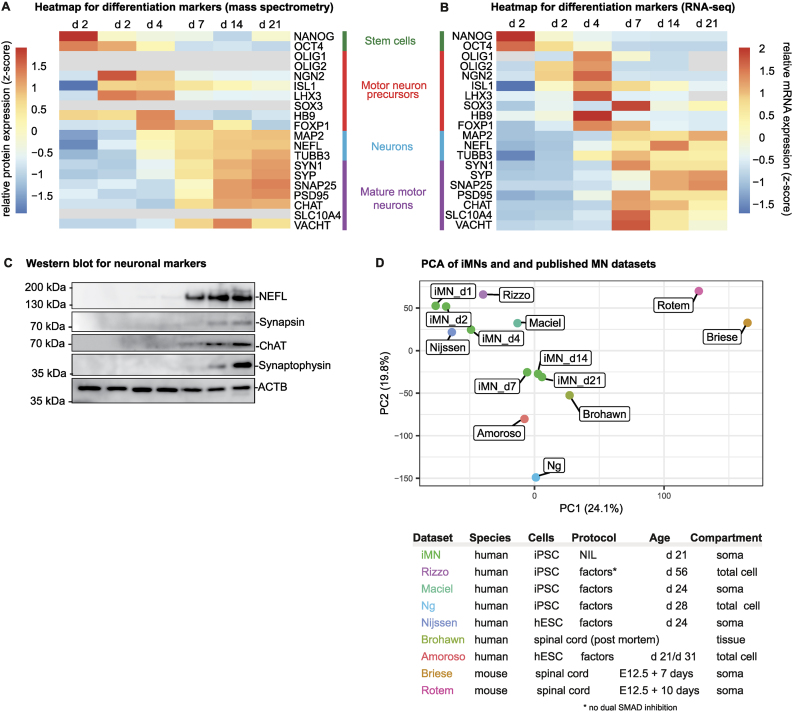
iMNs have MN marker signatures and are transcriptomically similar to primary MNs. (**A** and **B**) Heatmaps showing the expression of markers for stem cells (green), neuronal progenitors (red), neurons (blue), and mature MNs (magenta) along differentiation stages. Mass spectrometry values are shown in panel (A) and represent *z*-score transformed mean log2 LFQ. mRNA-seq values are shown in panel (B) and represents *z*-score transformed mean log2 TPM (transcripts per million of library reads). (**C**) Western blot of mature neuronal (NF, synapsin, and synaptophysin) and MN (ChAT) markers during differentiation. The position of size markers is indicated on the right. ACTB was used as a loading control. (**D**) iMNs are transcriptomically similar to primary cortical neurons. The principal component analysis of variance stabilization transformed (DESeq2::vsd) RNA-seq expression data from iMNs (this study) and publicly available datasets, presented in the table on the right. The table indicates the species, type of cells or tissues, age, subcellular compartment, and differentiation protocols used. “Factors” designates dual SMAD inhibition (unless marked*) and small molecule activation of the sonic hedgehog (SHH) pathway.

To better characterize the differentiation process, we performed mass spectrometry and RNA sequencing (RNA-seq) analyses of different differentiation stages (Fig. 2 and Supplementary Table S1). The proteomic and transcriptomic expression profiles of cell type markers, presented as heatmaps in Fig. 2A and B, show a clear progression from stem cells via neuronal precursors to mature MNs (see Supplementary Fig. S1 for global expression profiles).

Stem cell markers (*NANOG* and *OCT4*) are well expressed in stem cells (Fig. 2A and B, day 1) but were rapidly downregulated after doxycycline induction (days 2 and 4).

After doxycycline induction (day 2), MN-specific transcription factors *NGN2*, *ISL1*, and *LHX3* integrated into the NIL-hiPSC line become expressed (Fig. 2A and B). These factors induce other neuronal precursor markers (*OLIG1*, *OLIG2*, *SOX3*, and MN-specific *HB9*) and quickly subside after doxycycline is removed at day 4. These data identify days 2–4 as the neuronal precursor stage. Expression of the HB9 protein was also validated by imaging (Fig. 1D–E).

General neuronal markers MAP2, neurofilament (NEFL) and TUBB3 reach high expression levels by day 7, corresponding to early MN stage. Finally, mature neuronal markers become highly expressed from day 7 to 14 of differentiation, reaching the highest level by day 21 (Fig. 2A and B). Among those are components of synaptic vesicles: synapsin 1 (SYN1), synaptophysin (SYP), and synaptosomal-associated protein 25 (SNAP25), as well as postsynaptic density protein 95 (PSD95) with a scaffolding role at the postsynaptic compartment. Moreover, at these stages the cells expressed known markers of mature MNs: choline *O*-acetyltransferase (ChAT) [53] and sodium/bile acid cotransporter 4 (SLC10A4). ChAT is the enzyme responsible for the biosynthesis of the neurotransmitter acetylcholine at the cholinergic synapses. SLC10A4 is involved in loading of synaptic vesicles and co-localizes with ChAT in in cholinergic neurons [54, 55]. We validated the expression of mature MN and synaptic markers by western blotting (Fig. 2C). Indeed, expression of synaptic (NEFL, SYN1, and SYP) and mature MN markers (CHAT) reached the highest protein levels at day 21. Expression of these markers identifies the late stages of differentiation as mature MNs.

Next, we compared the transcriptome of our iMNs to the expression profiles of MNs in publically available transcriptomic datasets from primary and stem cell-derived MNs. For that, we used the same analysis pipeline [34] to process five RNA-seq datasets from human stem cell-derived MNs [36–38, 40, 42], one dataset from human spinal cord [39] and two datasets from mouse spinal cord or primary MNs [41, 43]. We compared the global similarities between the datasets using principal component analysis (PCA) (Fig. 2D). Critically, our iMNs (days 7–21) clustered together with the human spinal cord samples [39]. We detected more differences between spinal cords and MNs obtained with the differentiation protocols based on small molecules [36–38, 40, 42]. As expected, a clear separation between human and mouse data was observed in PCA. This analysis shows that our iMNs are transcriptomically similar to the mature human primary MNs and compare favorably to other available differentiation protocols in this regard.

### iMN are electrophysiologically active and form neuromuscular junctions

**Figure 3.**
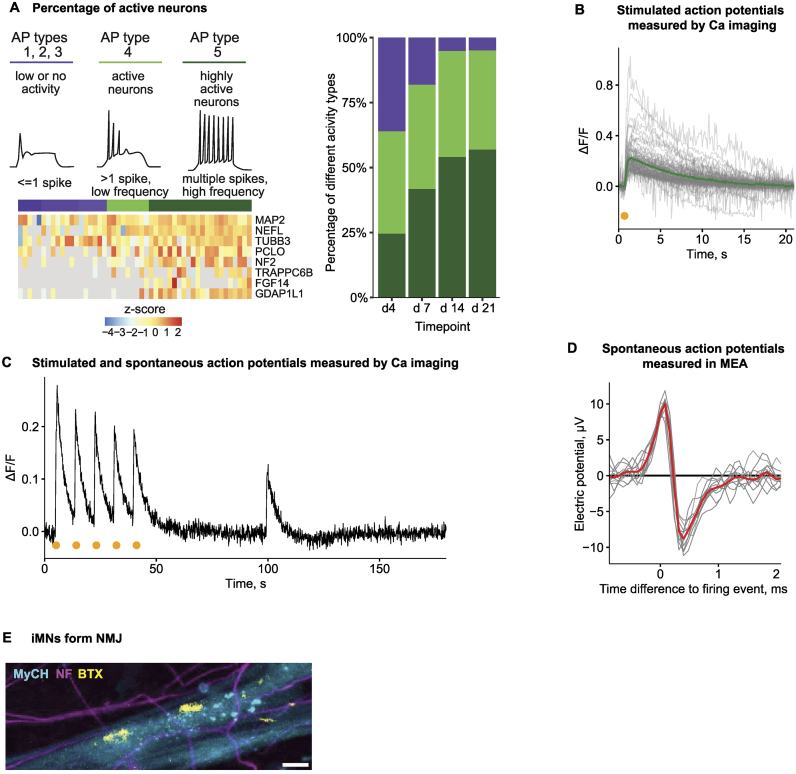
iMNs are electrophysiologically active. (**A**) Electrophysiological activity of iMNs, reflected in the frequency of spontaneous spikes, was predicted using scRNA-seq and electrophysiology data [45]. Left: Exemplary electrophysiology profiles of cells corresponding to different functional types (adapted from [45]) and heatmap showing *z*-score transformed expression values of general neuronal (MAP2, NEFL, and TUBB3), synaptic (PCLO and NF2) and active transmission markers (TRAPPC6B, FGF14, and GDAP1L1) used to determine the functional type. Right: Bar plot showing percentages of cells matched to each functional type for different differentiation stages. (**B** and **C**) Neuronal activity of iMNs recording with calcium imaging. (B) Calcium transients after induction of activity potentials in iMNs (day 20 after the start of differentiation) using 20 V stimulation (yellow mark). Fluorescence intensity changes from CalBryte520 are normalized to pre-stimulus baseline level, and individual measurements from 84 ROIs (gray) as well as the average (green) are shown. (C) Exemplary calcium transient of a single ROI over the 1 min induction and 2 min observation period. 20 V stimulation occurred at 5, 14, 23, 32, and 41 s time points (yellow marks), and changes in fluorescence intensity, normalized to baseline level, are shown. (**D**) APs measured in iMN grown in an Axion multiwell microelectrode array (MEA) plate. Multiple spikes from the same electrode as well as the average (red, *n* = 20) are shown. (**E**) iMNs form NMJs in co-cultures with myotubes. Axons are visualized with NF staining (magenta), myotubes—with α-myosin heavy chain (MyHC) staining (cyan), and NMJs—with BTX staining (yellow). Scale bar: 10 μm.

To ascertain the functionality of iMNs, we examined their expression profiles for the markers of electrophysiological activity. To achieve this, we used single-cell RNA-seq data from patch-clamped neurons, which associated expression profiles with different AP neuronal types [45, 46]. In particular, Bardy *et al.* [45] grouped cells based on their spike profiles in patch clamping experiments. Some cells showed single or no spike activity (Fig. 3A, left, low functionality: AP types 1, 2, 3, purple), other cells generated multiple spikes at low frequency (middle functionality: AP type 4, light-green), and there were also cells producing multiple spikes at high frequency (high functionality: AP type 5, dark-green). Groups with higher neuronal activity were correlated with upregulation of specific genes, for example, Piccolo (*PCLO*) required for neurotransmitter release at synapses [56], Trafficking protein particle complex subunit 6B (*TRAPPC6B*) involved in neuronal development [57], and others (Fig. 3A, left).

Relying on these data, we applied cell-type deconvolution [46] to our RNA-seq data produced from different differentiation stages of iMNs. This analysis showed that the functionality of iMNs increases as cells proceed through differentiation, with more than 90% of functional neurons (light- and dark-green bars) at day 21 (Fig. 3A, right).

We then experimentally measured the electrophysiological activity of iMNs using calcium imaging (Fig. 3B). Electrophysiological activity in neurons is accompanied by an influx of calcium ions, which can be measured with calcium indicators—fluorescent molecules that respond to the binding of calcium ions. Using electrical stimulation, we could evoke APs in mature iMNs, visualized with calcium transients after stimulation (Fig. 3B). Moreover, 67% of cells responding to electrical stimulation showed spontaneous activity potentials (Fig. 3C). In addition to calcium imaging, we used a multielectrode array (MEA, axion) to measure the electric activity of mature iMNs (Fig. 3D). These experiments confirmed that iMNs generate spontaneous APs with typical de- and repolarization patterns.

Another hallmark of functional mature MNs is the ability to form NMJs, specialized synapses between motor axons and muscle cells. To test if iMNs can form NMJs, we co-cultured them with muscle cells generated through the inducible expression of the myogenic transcription factor MyoD [58] using available protocols [22]. The functionality of these neuromuscular cultures has been confirmed by evidence of muscle contractions and their responsiveness to curare (Supplementary Movie S1), a drug that affects the interaction between motor axons and muscles [59]. To visualize NMJs, we stained the co-cultures for nicotinic acetylcholine receptors (nAchRs), which serve as a marker of NMJ. nAchR is recognized with a fluorophore-conjugated bungarotoxin (BTX, Fig. 3E). Indeed, we observed the formation of NMJs (BTX, yellow) at the sites of contact between motor axons (NF, magenta) and myotubes (myosin heavy chain, or MyCH, cyan). Thus, our analysis points to the hallmarks of functionality of iMNs—they exhibit electrophysiological activity and form functional NMJs.

### Efficient enrichment of motor axons for omics analyses

iMNs represent a promising cellular model to study MN diseases, such as ALS, spinal muscular atrophy (SMA), Charcot–Marie–Tooth disease (CMT), and others. Although it has been known for some time that the first signs of neurodegeneration in MN diseases manifest in axons [4–6, 60, 61], most prior studies in the field focused on whole neurons, therefore failing to detect local axonal changes. Therefore, we decided to adopt the spatial transcriptome and proteome analysis we developed earlier [15–19] to iMNs.

**Figure 4.**
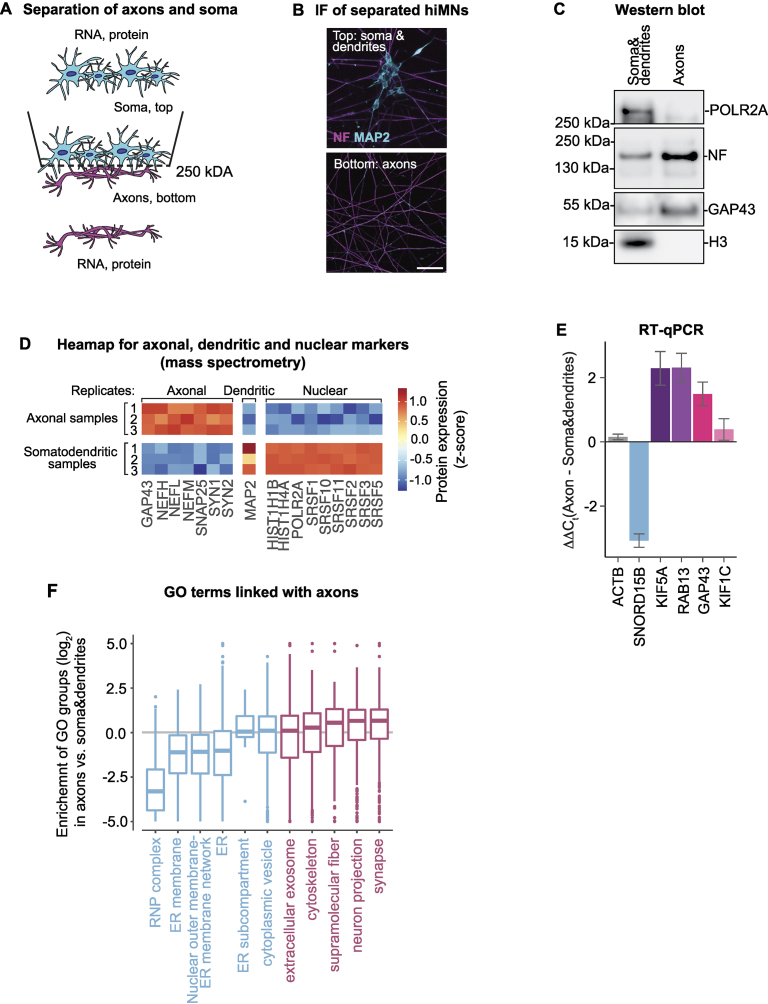
The local transcriptome and proteome of motor axons are linked with synaptic functions. (**A**) Scheme showing separation of iMNs on subcellular compartments. iMNs are grown on a microporous membrane with cell bodies (Soma) restricted to the top and axons extending to the bottom, allowing for the separation of the two compartments. (**B**) Exemplary image of iMNs grown on a microporous membrane: dendrites and soma (MAP2, cyan) are restricted to the top of the filter, while axons (NF, magenta) extend to the bottom. (**C**) Western blot analysis validating enrichment of somatic and axonal proteins in the separated compartments. Axons are enriched in NF and GAP43 and depleted of histone H3 and RNA polymerase II (POLR2A). Position and size markers are indicated on the left. (**D**) Heatmap showing expression of axonal, dendritic, and nuclear markers in three biological replicates of axonal and somatodendritic samples. Plotted and *z*-score transformed LFQ values obtained by mass spectrometry analysis. (**E**) qRT-PCR analysis validates the enrichment of somatic and axonal RNAs in separated subcellular compartments. Nuclear RNA *SNORD15B* is enriched in soma, while *ACTB* mRNA is equally distributed, and *RAB13*, *GAP43*, *KIF1C* and *KIF5C* show different degrees of enrichment in axons. The difference in expression levels (ΔΔ*C*_t_) of indicated transcripts between axonal and somatodendritic compartments samples is plotted on (*Y*). *18S* rRNA was used as a normalization control. Bar, mean ± SD, *n* = 3. (**F**) GO analysis indicates the enrichment of proteins with nuclear function in the soma and synaptic or neuronal functions in axons. Boxplots show the protein localization values (Axon/Soma log2 fold difference) of all proteins belonging to selected GO terms. Terms were selected from GO cellular compartments if they showed a significant overrepresentation of proteins and transcripts localized to either compartment in both the proteomic and transcriptomic data. See “Materials and methods” section for more details.

To separate iMNs into axons and soma, we grew them on a microporous membrane so that soma stayed on one surface, and axons extended through the pores on another membrane surface (Fig. 4A). We confirmed efficient separation by immunostaining of MNs growing on the membrane (Fig. 4B). Importantly, staining with axonal (NF) and dendritic (MAP2) markers confirmed that with this approach we can isolate motor axons, primarily affected in MN disease, rather than a mixture of axons and dendrites. We also validated the enrichment of selected axonal (NF and GAP43) and somatic markers (histone H3 and RNA II polymerase POLR2A) by western blotting on isolated subcellular compartments (Fig. 4C).

We then proceeded to isolate axons and somatodendritic compartments from either side of the membrane. To identify their local proteomes and transcriptomes, we performed liquid chromatography-tandem mass spectrometry (LC-MS/MS) and RNA-seq of isolated subcellular compartments. We measured 5418 proteins using a LFQ method [32] and 15 610 transcripts using RNA-seq (Supplementary Tables S2 and S3, see Supplementary Fig. S2A–D for quality controls). We performed differential expression analysis to identify proteins and transcripts that are differentially localized between two subcellular compartments. We defined axon- and soma-localized proteins and mRNAs based on their fold enrichment in axons versus somatodendritic compartment: axon-localized proteins and mRNAs are defined as significantly enriched in axons by at least 2-fold (Supplementary Fig. S2E and F, magenta), and soma-localized mRNAs (cyan), as enriched in somatodendritic compartment according to the same criterion. Importantly, analysis of three biological replicates of isolated compartments confirmed consistent enrichment of axonal markers in axonal samples and dendritic (MAP2) and nuclear markers—in somatodentritic samples (Fig. 4D), additionally validating our ability to enrich the axonal fraction. Among axonal markers were neurofilaments (NEFL, NEFM, and NEFH), a major component of axonal growth cones neuromodulin (growth-associated protein 43, or GAP43 [62]), components of synaptic vesicles synapsins 1–3 (SYN1, SYN2, and SYN3) and SNAP25 [63]. Nuclear markers included histones (HIST1H1B, HIST1H3A, and HIST1H4A), a subunit of RNAII polymerase (POLR2A), and splicing factors (SRSF1, SRSF2, SRSF3, SRSF5, SRSF10, and SRSF11).

Additionally, at the mRNA level, transcripts encoding several axonal markers were enriched in axons (Supplementary Fig. S2F, magenta). These included *Gap43* [62], kinesin motor proteins involved in axonal transport such as kinesin-like protein KIF1C (*Kif1c*) [64] and kinesin heavy chain isoform 5A (*Kif5a*) [65], which has previously been reported to be axonally enriched [66] and a major regulator of neurodegeneration [67]; *Rab13*, encoding a component of transport vesicles co-localizing with GAP43 [68], enriched in neurites across multiple types of neurons [69, 70]. We validated the axonal enrichment of these transcripts by reverse transcription (RT)-qPCR on RNA isolated from motor axons and soma (Fig. 4E, purple to red bars). In contrast, nuclear marker small nucleolar RNA 15b (*Snord15b*, blue bar) was somatically enriched in our RT-qPCR analysis.

To functionally characterize local proteomes and transcriptomes of iMNs we performed GO overrepresentation analysis on proteins and transcripts enriched in one of the subcellular compartments (Fig. 4F, Supplementary Table S4). We then plotted an average enrichment in axons versus soma for all annotated proteins in each overrepresented GO term in the category “cellular compartments”. This analysis showed that axonal proteome and transcriptome are associated with neuronal development and synaptic components, while somatic proteome and transcriptome—with the nuclear and endoplasmic reticulum (ER) components. Thus, our combined experiments demonstrate the feasibility of efficient separation of iMN-derived subcellular compartments for functional omics analyses.

### FUS^R244RR^–ALS–hiPSC model recapitulates neurodegenerative phenotypes

**Figure 5.**
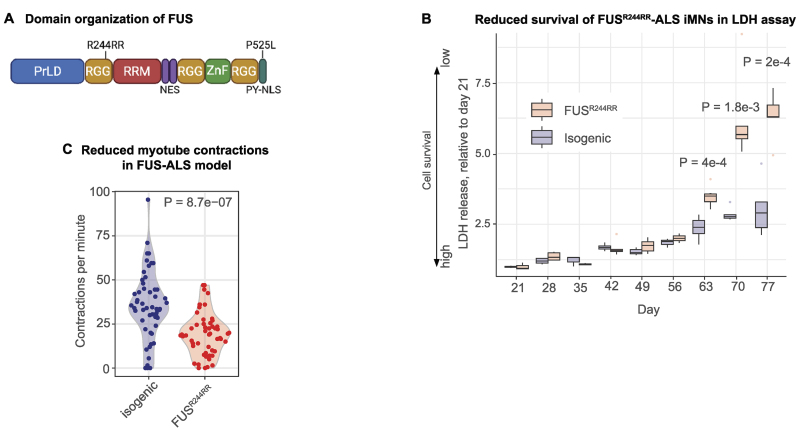
FUS^R244RR^–ALS hiPSC models recapitulate patient phenotypes. (**A**) Domain structure of FUS protein, including the positions of the examined R244RR mutation and the frequently researched P525L mutation (reviewed in [71]). PrLD: prion-like domain involved in FUS dimerization, RRG: arginine-glycine-glycine-rich domains, RRM: RNA recognition motif, NES: nuclear export signals, ZnF: zink-finger motif, PY-NLS: nuclear localization signal. RRM, ZnF, and RGG participate in RNA and protein binding. (**B**) The LDH release assay shows lower survival of FUS^R244RR^ MNs (red) compared with isogenic control (blue). Boxplots showing LDH release, reflecting the level of plasma membrane damage (Y) at different time points (X). T-test *P*-values for days 63, 70, and 77 are shown on the plot. (**C**) The number of myotube contractions per minute (X) is plotted for FUS-ALS MNs (red) and control isogenic MNs (blue). *P*-value was calculated using *t*-test.

To determine if iMNs can replicate the neurodegenerative phenotypes observed in ALS, we established a NIL-hiPSC line from the fibroblasts of an ALS patient with a mutation *FUS* gene (NIL–FUS^R244RR^–hiPSC). FUS is an RBP playing a role in splicing and nucleo-cytosolic RNA transport and translation (reviewed in [71]). FUS^R244RR^ has a mutation in the RGG domain (Fig. 5A), and mutations in this position have been reported to accelerate the conversion of FUS to a fibrous state, an event linked with disease [72]. As a control, we created an isogenic line with the corrected FUS mutation (NIL-iso-hiPSC). We then differentiated these lines into iMNs and assessed their viability through a lactate dehydrogenase (LDH) assay. LDH is a cytosolic enzyme released into the cell culture medium upon loss of membrane integrity. LDH levels in the medium can be measured with a colorimetric assay. Our LDH measurements showed a ∼2.3-fold higher cell death of FUS^R244RR^–iMNs compared with the negative control isogenic iMNs (Fig. 5B), pointing to the recapitulation of neurodegenerative phenotype in our test system.

ALS is characterised by progressive muscular paralysis reflecting degeneration of motor axons. To evaluate the progression of this degeneration in our ALS model, we used neuromuscular cultures. For that, we co-cultured either FUS^R244RR^–ALS iMNs or isogenic control iMNs with muscle cells derived from hiPSCs through the inducible expression of MyoD [58]. We then counted the number of contractions in each co-culture. Notably, the FUS^R244RR^–ALS cultures exhibited a significantly reduced number of contractions compared to the isogenic controls, mirroring the impairment observed in patients (Fig. 5C; Supplementary Table S5 and Supplementary Movie S2). This analysis confirms that our neuromuscular cultures successfully recapitulate patient-specific phenotypes and can be effectively used to study the molecular mechanisms behind observed neurodegeneration defects.

### Spatial omics of hiPSC-derived ALS models

**Figure 6.**
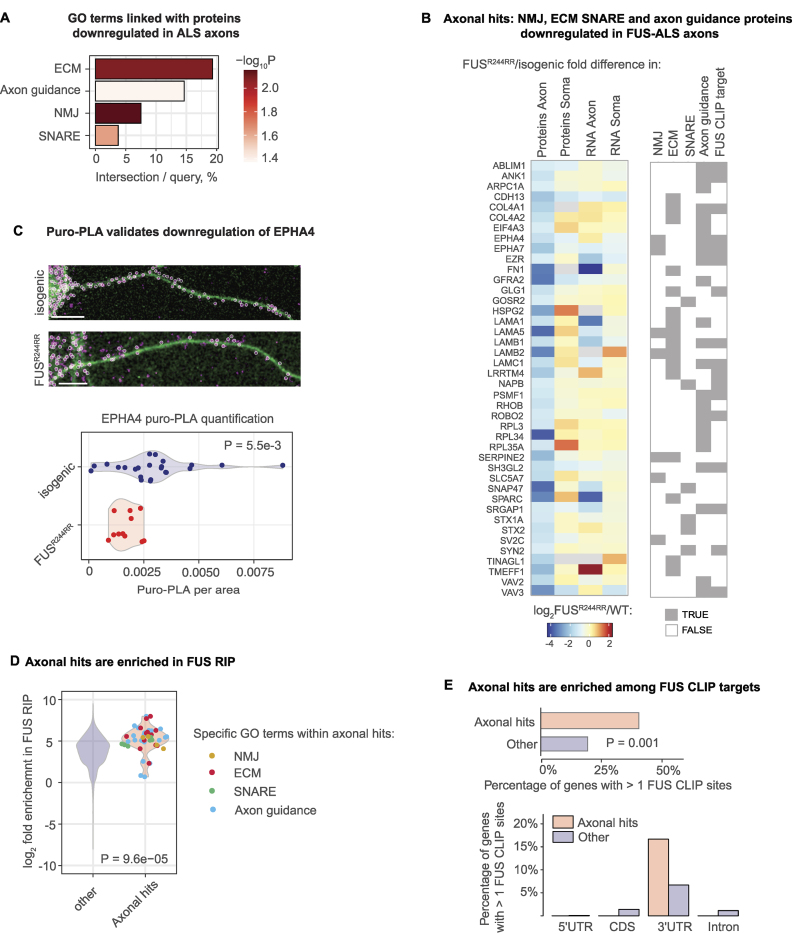
Proteins downregulated in FUS^R244RR^–ALS axons are linked in NMJ functionality and enriched in FUS targets. (**A**) GO terms associated with proteins downregulated in axons of FUS^R244RR^–ALS versus isogenic iMNs. NMJ, ECM, SNARE complex, involved in presynaptic membrane trafficking. (**B**) Heatmap showing changes in expression of proteins and RNAs belonging to downregulated GO term presented in panel (A) between FUS^R244RR^–ALS and isogenic iMNs (log2 fold difference). The protein and RNA expression levels were measured by mass spectrometry and mRNA-seq analyses of axons and cell bodies. Specific GOs for each gene are indicated below the heatmap. This group of genes is further referred to as axonal hits. (**C**) Validation of EPHA4 downregulation in FUS^R244RR^–ALS versus isogenic iMNs with puro-PLA. Representative EPHA4-puro-PLA images of FUS^R244RR^–ALS and isogenic iMNs (above) and quantification of puro-PLA signal (below). EPHA4-puro-PLA: magenta; NF (serving to outline cell borders): green, scale bar: 10 μm. Circled are EPHA4-puro-PLA spots used for quantification. Quantification: Violin plots showing EPHA4-puro-PLA signal normalized per area in axons of FUS^R244RR^-ALS (red) versus isogenic (blue) iMNs. Individual datapoints correspond to single neurons (*n*(FUS^R244RR^-ALS) = 11, *n*(isogenic) = 24). *P*-value was calculated using *t*-test. (**D**) Axonal hits are enriched among FUS RIP targets. Violin plots showing fold enrichment (log2) of axonal hits (red) and all other RNAs (blue) in FUS RIP. FUS RIP data from [82] were used. Enrichment of indivudual RNAs within axonal hits is shown with a scatterplot and specific GO terms are indicated with color (NMJ: beige, ECM: red, SNARE: green, axon guidance: blue). (**E**) Axonal hits are enriched among FUS CLIP targets. Top: Barplot showing the percentage of FUS CLIP targets (>1 CLIP site) among axonal hits (red) and other transcripts (blue). *P*-value computed using Chi squared test is shown. Bottom: Barplot showing the distribution of FUS CLIP sites among different gene regions (5′UTR, CDS: coding sequence, 5′UTR, introns) for axonal hits (red) and other transcripts (blue). *P*-value computed using proportion test for 3′UTRs is shown. FUS CLIP data are taken from [83].

Next, we analyzed the changes in the local proteome of FUS^R244RR^–iMNs that could explain the reduced survival and functionality of mutant iMNs. We performed GO enrichment analysis of proteins differentially expressed in FUS^R244RR^ axons compared with isogenic axons (Supplementary Table S6). Curiously, we detected axonal downregulation of proteins linked with NMJ, extracellular matrix (ECM), soluble N-ethylmaleimide-sensitive factor activating protein receptor (SNARE) complex, and axon guidance (Fig. 6A, see Supplementary Table S7 for more details). These components are crucial for synapse and NMJ structure and plasticity (reviewed in [73]), and for simplicity, we refer to these proteins as axonal hits. The axonal hits include, for example, the Ephrin receptor EPHA4, a component of NMJ [74] whose knockout causes motor deficits in mice [75]; the choline transporter SLC5A7, mutated in hereditary motor neuropathies [76]; laminin A (LAMA1) whose neuronal knockdown results in abnormal morphology and neurite formation [77]; laminin B (LAMB1) whose mutation in mice leads to dystonia-like movement disorders with spinal defects [78]; and heparan sulfate proteoglycan 2 (HSPG2), which is aberrantly spliced in MNs of ALS patients [79] and associated with tardive dyskinesia [80] (Fig. 6B). Additionally, SNARE proteins, downregulated in FUS^R244RR^ axons, are crucial for the docking and fusion of synaptic vesicles with the plasma membrane (reviewed in [81]) and thus are important for signal transmission to muscle cells (Fig. 6B). These changes in the axonal proteome offer a mechanistic explanation for the observed functional defects in neuromuscular function, as detected in our contractility assay (Fig. 5C). Representative images of mutant and wild-type neurons stained for axonal (NF) and dendritic (MAP2) markers (Supplementary Fig. S3A) show no major morphological differences between the two groups. This suggests that the observed axonal proteome changes are not simply a consequence of altered neuron morphology but rather reflect intrinsic molecular dysfunction.

Curiously, most axonal hits showed downregulation only in axons or stronger downregulation in axons than in cell bodies (Fig. 6B). Such changes thus could not be detected with an analysis of whole MNs, so this experiment provides a proof of principle for the importance of subcellular analysis in ALS.

To understand if this downregulation is restricted to proteins or affects both proteins and mRNAs encoding them, we performed RNA-seq analysis of isolated subcellular compartments (Supplementary Table S8). mRNAs of 30% of axonal hits (13 out of 42) showed downregulation in ALS motor axons; similarly to proteins, these transcripts were downregulated only in axons or more downregulated in axons than in cell bodies (Fig. 6B). For the remaining 70%, the changes at the protein level could not be explained by changes in the levels of mRNAs encoding them (Fig. 6B), suggesting that translation or protein localization may be affected. For example, NMJ component EPHA4 [74] is downregulated in FUS^R244RR^–ALS axons at the level of protein by ∼2-fold, but its mRNA levels are not significantly affected (Fig. 6B). We therefore decided to test if the translation of EPHA4 is affected in FUS^R244RR^–ALS axons. We visualized its translation by the puro-PLA assay [25], which relies on puromycin-tagging of newly synthesized proteins. Our results demonstrated that EPHA4 translation is indeed downregulated in FUS^R244RR^ axons compared with the isogenic control (Fig. 6C).

Next, we sought to understand the mechanism behind the downregulation of these proteins in FUS^R244RR^ axons. FUS is an RBP that plays a role in multiple steps of RNA metabolism, including splicing, nucleo-cytosolic RNA transport, and translation (reviewed in [71]). To explore if FUS may downregulate axonal hits by binding directly to transcripts encoding them, we analyzed data of FUS RNA-immunoprecipitation (RIP) from human cells [82] Supplementary Table S9). Our analysis revealed that axonal hits, presented in Fig. 6B, are enriched in FUS RIP (Fig. 6D).

CLIP (crosslinking immunoprecipitation) offers a significant advantage over RIP in that it enables the detection of RBP-binding sites on RNA, thus allowing for the identification of direct RNA targets. We therefore also utilized FUS CLIP data ([83] and Supplementary Table S10). The Chi-squared test revealed a statistically significant enrichment of FUS CLIP sites among axonal hits (Fig. 6E). Specifically, 40% of axonal hits contained more than one FUS CLIP site (17 out of 42), which was a 2-fold enrichment over other transcripts, in which 19% had more than one FUS CLIP site. Notably, most of FUS CLIP sites in axonal hits were located within 3′UTRs (Fig. 6E). Moreover, “synapse organization” emerged as the top GO term enriched among axonal hits carrying FUS CLIP sites (Supplementary Fig. S3B). Together, the FUS RIP and CLIP data suggest that a substantial part of downregulated genes may represent direct FUS targets.

## Discussion

As the global population ages, neurodegenerative diseases are projected by the World Health Organization to become the second leading cause of death by 2040, surpassing cancer-related deaths (reviewed in [1]). Current therapeutic strategies primarily target specific genetic mutations responsible for these diseases. For example, antisense oligonucleotides (ASOs) are used to target and degrade aberrant mRNAs associated with various neurodegenerative diseases, such as ALS (targeting SOD1 and C9ORF72) (reviewed in [84, 85]). However, this approach has limitations due to the heterogeneous etiology of many neurodegenerative diseases. ALS, for instance, is linked to mutations in over 40 different loci, with SOD1 and C9ORF72 mutations accounting for only about 10% of all ALS cases (reviewed in [86]). Moreover, for the majority of ALS patients, no traceable genetic cause can be identified, making targeted ASO therapies unsuitable for most cases. This highlights the necessity of understanding common defects across ALS cases of different etiologies.

Neurons are highly polarized cells with unique morphologies, comprising a cell body and neurite extensions—axons and dendrites. Axons play critical roles in conducting electrical signals and can be very long. Due to this specialized structure, many cellular components, including RNAs, proteins, and organelles, need to be transported into axons, where transported mRNAs are translated to produce local proteins (reviewed in [87]). This distinctive morphology is not found in any other cell type in the body and contributes to the vulnerability of neurons to neurodegenerative diseases. Compelling evidence shows that axonal loss is an early and predominant feature in multiple neurodegenerative diseases, preceding clinical symptoms and occurring before cell body degeneration (reviewed in [3]). This early event in the pathological process offers a potential therapeutic target.

Here, we established a new hiPSC-based model for ALS to study the mechanisms behind axonal dysfunction. We generated a hiPSC line from the fibroblasts of an ALS patient with an R2444RR mutation in the RGG domain of the *FUS* gene (FUS^R244RR^, Fig. 5A). This is the first FUS-ALS model featuring this mutation. Although not identical, a mutation at the same position (R244C) has been studied *in vitro* and reported to accelerate the conversion of FUS droplets to a fibrous state [72]. This model is crucial because most prior studies focused on FUS mutations affecting the NLS, causing protein mislocalization to the cytoplasm and leading to early disease onset.

Determining whether mutations in the RGG domain are causative for ALS is challenging due to limited Genome-Wide Association Study (GWAS) data, which systematically links genetic variants with diseases across populations (reviewed in [88]). This limitation underscores the importance of hiPSC-based models like ours, which allow us to analyze functional and molecular phenotypes in patient-derived cells compared to isogenic controls where the mutation is corrected. Studies like this are increasingly valuable as researchers work to clarify the pathogenic role of mutations outside the well-characterized FUS NLS mutations. By expanding the diversity of ALS models, our work contributes to a deeper understanding of disease pathogenesis.

We demonstrated that this model recapitulates phenotypes typical of ALS patients, such as reduced cell viability and reduced neuromuscular contractility (Fig. 5B and C). Additionally, we developed an approach to efficiently isolate axonal and somatodendritic compartments from hiPSC-derived MNs for omics analysis. This analysis revealed a preferential downregulation of proteins linked with NMJ, ECM, SNARE complex, and axon guidance in FUS–ALS axons, which we termed axonal hits (Fig. 6A and B). The axonal hits include, for example, the Ephrin receptor EPHA4, which mediates the phosphorylation of the actin-binding protein cortactin, thereby playing a role in NMJ maintenance [74]. Consistently, EPHA4 knockout causes motor deficits in mice [75]. Another hit is the choline transporter SLC5A7, which mediates choline uptake, a precursor of the MN neurotransmitter acetylcholine, and is mutated in hereditary motor neuropathies [76].

Crucially, these changes could not have been detected if the analysis had been performed on whole MNs, highlighting the importance of a subcellular analysis. Given the crucial role of these components in synapse and NMJ structure and plasticity, changes in the axonal proteome provide a mechanistic explanation for the observed neuromuscular functional defects detected in our contractility assay (Fig. 5C). The exact mechanism by which mutant FUS leads to this dysregulation remains a key question for future studies.

Analysis of FUS RIP and CLIP data revealed that the FUS targets are enriched among the axonal hits, suggesting that many of these transcripts are directly regulated by FUS. Considering the involvement of FUS in RNA splicing (reviewed in [71]), we investigated whether the splicing of these transcripts is altered in FUS^R244RR^ iMNs. However, we found no splicing changes that could account for the altered expression of axonal hits in FUS^R244RR^–ALS (Supplementary Table S11). Most axonal hits are affected at the protein level but not at the RNA level (Fig. 6B), suggesting they are translationally downregulated in FUS^R244RR^–ALS axons. FUS is a multifunctional RBP known to influence multiple levels of gene expression, including translation (reviewed in [71]).

For instance, FUS has been reported to promote the translation of mRNAs localized to cellular protrusions in mouse fibroblasts with the assistance of the adenomatous polyposis coli (APC) protein [89]. Conversely, ALS-linked FUS mutants were found to repress translation by forming heterogeneous condensates with fragile X mental retardation protein (FMRP) and sequestering bound RNAs from the translational machinery [90]. Similarly, another study reported that mutant FUS sequesters proteins associated with translation and RNA quality surveillance pathways, thereby suppressing protein translation [91]. Additionally, ALS-linked FUS mutants have been shown to associate with stalled polyribosomes and reduce global protein synthesis [92]. Activation of the integrated stress response (ISR) has also been reported in FUS–ALS neurons [93]. ISR is a key regulatory mechanism activated by various stress conditions (reviewed in [94]). It is triggered by the phosphorylation of the alpha subunit of eukaryotic initiation factor 2 (eIF2α), which in turn reduces the levels of the ternary complex eIF2:GTP:Met-tRNA^i^ required for translation initiation, leading to global translational inhibition. Notably, we have not detected an increase in eIF2α phosphorylation in our FUS–ALS model (data not shown). However, our model represents a mild form of ALS, with a mutation in the RGG domain rather than a mutation in the NLS domain, which results in early-onset ALS and has been the focus of the prior studies cited above.

In summary, we describe a novel mutation and establish a new patient-derived iPSC ALS model, demonstrate its impact on axonal gene expression, and provide a molecular explanation for the observed phenotype. Applying this methodology to other neurodegenerative models could elucidate shared pathways and molecular targets, paving the way for the development of broad-spectrum therapies aimed at restoring cellular functions and halting disease progression. This could result in more effective and accessible treatments, reducing the reliance on personalized therapies.

## Supplementary Material

ugaf005_Supplemental_Files

## Data Availability

The mass spectrometry proteomics data have been deposited to the ProteomeXchange Consortium via the PRIDE partner repository with the dataset identifier PXD031066 and PXD054876. Raw RNA-seq data have been deposited to EBI ArrayExpress with identifiers E-MTAB-14352, E-MTAB-14354, and E-MTAB-14359. The pipeline for puro-PLA data analysis can be found at https://github.com/LauraBreimann/ALS_puro-PLA. Supplementary movies are deposited at Figshare with doi 10.6084/m9.figshare.26412352 (Supplementary Movie S1) and 10.6084/m9.figshare.26503363 (Supplementary Movie S2). The code was deposited to Zenodo (doi: 10.5281/zenodo.14293534) and Figshare (doi: 10.6084/m9.figshare.28418357).
