# Supplementary material for: Neuromuscular dysfunction in patient-derived FUSR244RR–ALS iPSC model via axonal downregulation of neuromuscular junction proteins
Source: NAR Mol Med. 2025 Mar 12;2(2):ugaf005. doi: 10.1093/narmme/ugaf005 (PMC12429959; doi:10.1093/narmme/ugaf005)
Supplement: ugaf005_Supplemental_Files [file ugaf005_Supplemental_Files.zip › List of Supplementary tables.pdf]

List of Supplementary tables:

Table S1. Mass spectormetry and RNA sequencing data for different differentiation stages of iMNs.

Table S2. Mass spectormetry data for axonal and somatodendritic compartments of iMNs.

Table S3. RNA-seq data for axonal and somatodendritic compartments of iMNs.

Table S4. Gene Ontology analysis of differentially expressed proteome in axonal versus somatodendritic compartments

Table S5. Contractions in FUS(R244RR) and isogenic control in neuromuscular cultures.

Table S6. Mass spectormetry data for axonal and somatodendritic compartments of FUS(R244RR) and isogenic control iMNs.

Table S7. Gene Ontology analysis of differentially expressed axonal proteome in FUS(R244RR) versus isogenic control iMNs.

Table S8. RNA-seq data for axonal and somatodendritic compartments of FUS(R244RR) and isogenic control iMNs.

Table S9. FUS RNA Immunoprecipitation data (82).

Table S10. FUS CLIP data (83).

Table S11. Differential splicing analysis in FUS(R244RR) and isogenic control iMNs.
