# Supplementary material for: Neuromuscular dysfunction in patient-derived FUSR244RR–ALS iPSC model via axonal downregulation of neuromuscular junction proteins
Source: NAR Mol Med. 2025 Mar 12;2(2):ugaf005. doi: 10.1093/narmme/ugaf005 (PMC12429959; doi:10.1093/narmme/ugaf005)
Supplement: ugaf005_Supplemental_Files [file ugaf005_Supplemental_Files.zip › Supplementary figures.pdf]

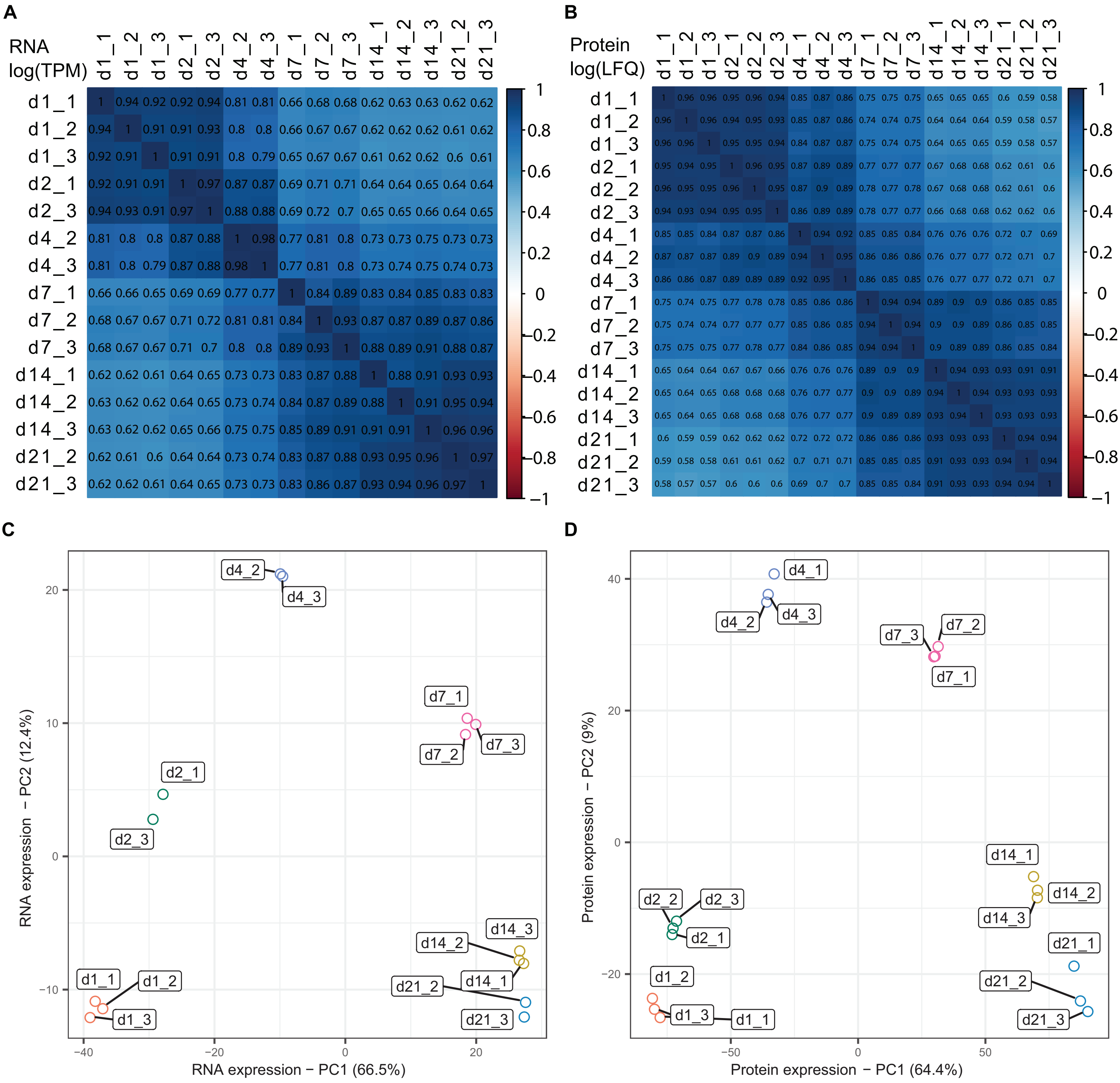

Figure S1. Quality control plots for transcriptomic and proteomic data from iMN time course experiment.

(A-B) Correlation plots of expression of RNA (A, log10(TPM), with 0.01 pseudocount) and protein (B, logLFQ) in different samples taken from iMN at different stages of differentiation. The number shown in each rectangle represents the Pearson correlation coefficient between the samples.

(C-D) The principal component analysis (PCA) of iMN expression data from different timepoints. PCA was calulated using only genes/proteins detected in all samples based on (C) log10(TPM) and (D) logLFQ values.

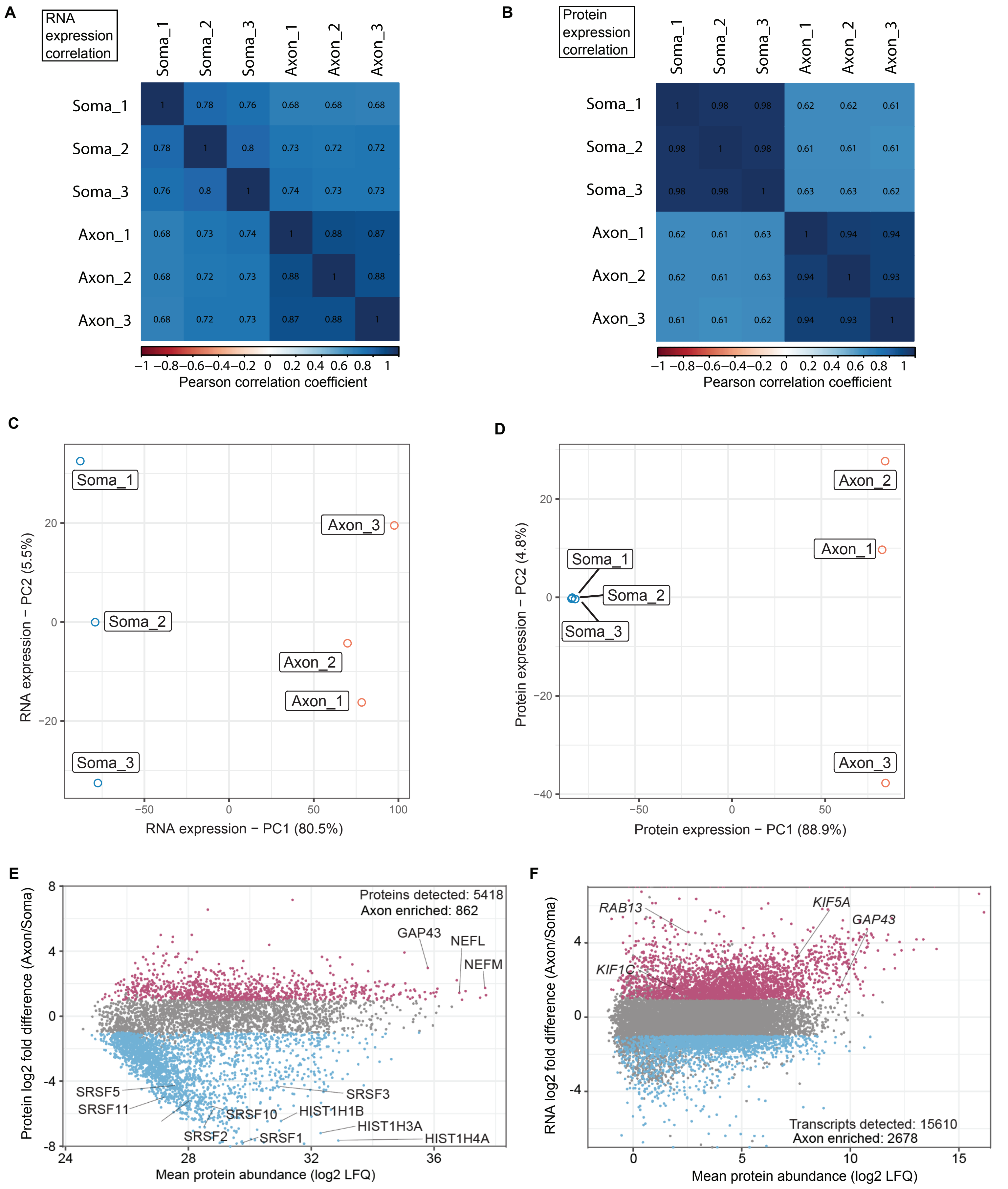

Figure S2. Quality control plots for transcriptomic and proteomic data of separated iMN compartments.

(A-B) Correlation plots of expression of RNA (A, log10(TPM), with 0.01 pseudocount) and protein (B, logLFQ) in axonal and somatodendritic compartments from iMNs at day 21 of differentiation. The number shown in each rectangle represents the Pearson correlation coefficient between the samples.

(C-D) The principal component analysis of axon and soma compartment from iMN expression data at day 21 after differentiation. PCA was calculated using only genes/proteins detected in all samples based on (C) log10(TPM) and (D) logLFQ values.

(E-F) MA plots showing the distribution of log2 fold difference values of proteins (E) and RNA (F) between axonal and somatodendritic compartments in relation to mean abundance (log2LFQ for proteins and log2TPM for RNA). Color indicates significant enrichment (p.adj < 0.05 & |log2fd| > 1) in either compartment (axons: purple; soma&dendrites: blue).

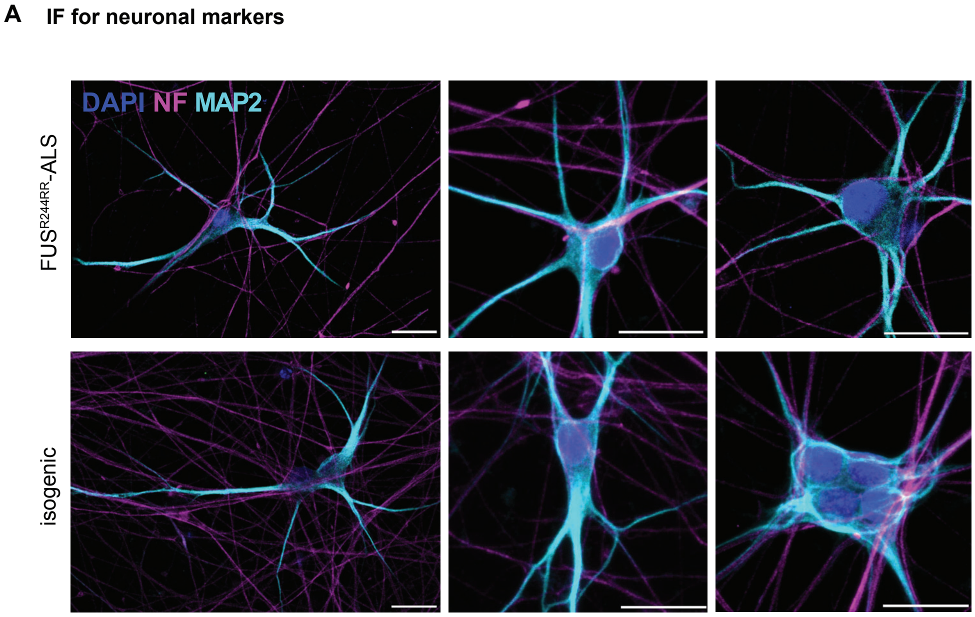

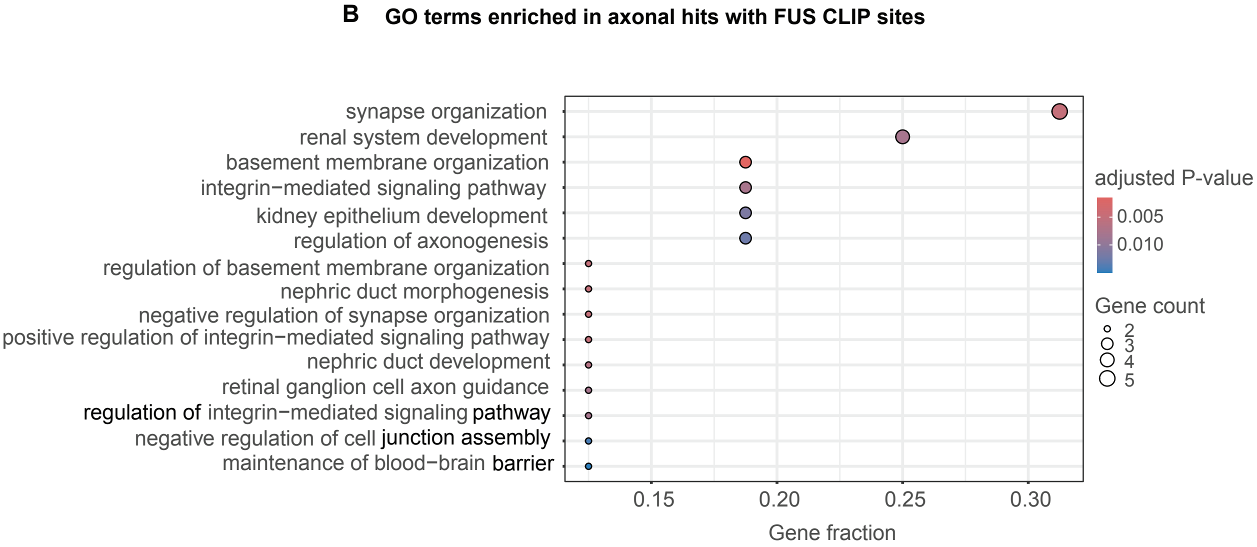

Figure S3. Morphology of FUS^{R244RR}-ALS and control iMNs, and GO terms enriched in axonal hits downregulated in FUS^{R244RR}-ALS axons.

(A) No major morphological differences were observed between FUS^{R244RR}-ALS and control iMNs. Representative images show mutant and control iMNs stained with antibodies against axonal (Neurofilament; NF, magenta) and dendritic (MAP2, cyan) markers. Blue: DAPI. Scale bar = 20 µm.

(B) GO biological process terms enriched in the 17 axonal hits with >1 FUS CLIP clusters. For each GO term listed on the Y-axis, the X-axis shows the fraction of genes involved in that term. The size of each dot corresponds to the number of genes associated with the given GO term, while the color represents the adjusted p-value.
